# Fluid-Mediated Stochastic Self-Assembly at Centimetric and Sub-Millimetric Scales: Design, Modeling, and Control

**DOI:** 10.3390/mi7080138

**Published:** 2016-08-06

**Authors:** Bahar Haghighat, Massimo Mastrangeli, Grégory Mermoud, Felix Schill, Alcherio Martinoli

**Affiliations:** 1 Distributed Intelligent Systems and Algorithms Laboratory (DISAL), School of Architecture, Civil and Environmental Engineering, École Polytechnique Fédérale de Lausanne (EPFL), EPFL ENAC IIE DISAL, Building GR, Station 2, 1015 Lausanne, Switzerland; felix.schill@epfl.ch (F.S.); alcherio.martinoli@epfl.ch (A.M.); 2 Physical Intelligence Department, Max Planck Institute for Intelligent Systems, Heisenbergstr. 3, 70569 Stuttgart, Germany; mastrangeli@is.mpg.de; 3 Cisco Systems, Av. des Uttins 5, 1180 Rolle, Switzerland; gmermoud@cisco.com

**Keywords:** self-assembly, stochastic systems, modeling, control

## Abstract

Stochastic self-assembly provides promising means for building micro-/nano-structures with a variety of properties and functionalities. Numerous studies have been conducted on the control and modeling of the process in engineered self-assembling systems constituted of modules with varied capabilities ranging from completely reactive nano-/micro-particles to intelligent miniaturized robots. Depending on the capabilities of the constituting modules, different approaches have been utilized for controlling and modeling these systems. In the quest of a unifying control and modeling framework and within the broader perspective of investigating how stochastic control strategies can be adapted from the centimeter-scale down to the (sub-)millimeter-scale, as well as from mechatronic to MEMS-based technology, this work presents the outcomes of our research on self-assembly during the past few years. As the first step, we leverage an experimental platform to study self-assembly of water-floating passive modules at the centimeter scale. A dedicated computational framework is developed for real-time tracking, modeling and control of the formation of specific structures. Using a similar approach, we then demonstrate controlled self-assembly of microparticles into clusters of a preset dimension in a microfluidic chamber, where the control loop is closed again through real-time tracking customized for a much faster system dynamics. Finally, with the aim of distributing the intelligence and realizing programmable self-assembly, we present a novel experimental system for fluid-mediated programmable stochastic self-assembly of active modules at the centimeter scale. The system is built around the water-floating 3-cm-sized Lily robots specifically designed to be operative in large swarms and allows for exploring the whole range of fully-centralized to fully-distributed control strategies. The outcomes of our research efforts extend the state-of-the-art methodologies for designing, modeling and controlling massively-distributed, stochastic self-assembling systems at different length scales, constituted of modules from centimetric down to sub-millimetric size. As a result, our work provides a solid milestone in structure formation through controlled self-assembly.

## 1. Introduction

Self-assembly (SA) plays a key role in many of the natural structuring phenomena at all scales. SA is defined as the reversible and spontaneous phenomenon of an ordered spatial structure emerging from the aggregate behavior of simpler preexisting building blocks through inherently local interactions in the system. The process of SA can be controlled through the proper design of the building blocks, the environment and the interplay of the involved forces. Several classes of SA can be distinguished, in particular static, dynamic and programmable SA [1]. In static SA, structures are formed at thermodynamic equilibrium, as the system reaches either a local or global equilibrium via energy minimization. The realm of static SA has been highly studied in engineered self-assembling systems [2,3,4]. In dynamic SA, stable non-equilibrium structures are formed and their persistence is accompanied by the dissipation of energy in the system. In programmable SA, information about the topology or functionality of the target structure is encoded in the chemical, physical or logical characteristics of the building blocks.

During recent years, SA has been extensively studied both as an enabling technique for micro-/nano-fabrication and as a coordination mechanism for distributed robotic systems of miniaturized modules with limited capabilities. Achieving the level of scalability and robustness, which inherently exists in natural self-assembling instances, both in terms of swarm sizes and the size of individual building blocks, has been a key motivation for investigating SA in engineered collective systems, where typically multiple simple and small building blocks are deployed. Simplicity in the building blocks’ internal design allows for reducing costs, increasing robustness and also further reduction of the blocks’ size, ultimately resulting in finer resolutions in the assembled target structures. With the recent advances in micro- and nano-engineering, the robotics community is now envisioning highly miniaturized robots roaming in environments inaccessible to conventional robotic platforms and, thus, holding great promises in a vast variety of domains, including biomedical engineering, pervasive information technology and environmental engineering [5,6]. At small physical scales, computation, sensing, actuation and communication capabilities become severely restricted. In addition, inherently highly noisy interactions are unavoidable at these scales. In order to mitigate these difficulties, stochastic coordination approaches can be leveraged. Such approaches allow distributed robotic systems to perform reliably and to accomplish tasks beyond the capabilities of a single robot.

An abundant body of research in swarm robotics has addressed the study and design of distributed, stochastic control strategies that are scalable in two senses: first, in terms of the swarm size (i.e., feasible in swarms of thousands of robots or more [7]) and, second, in terms of the individual module size (i.e., feasible on miniature robots with very limited capabilities [8]). Within the broader perspective of investigating how one can transpose and adapt such control strategies from the centimeter-scale down to the (sub-)millimeter-scale and from mechatronic to MEMS-based technology, our research efforts during the past few years have aimed to study and employ SA as a general methodology to achieve temporal and spatial coordination in distributed systems whose individual constitutive modules might be endowed with a varying level of complexity. Our research comprises two main thrusts: (i) a technological thrust focusing on the design, fabrication and packaging of experimental platforms for stochastic fluidic SA constituted of modules of different complexity and length scales; and (ii) a theoretical thrust focusing on developing stochastic control schemes supported by a flexible modeling framework. In particular, we have developed flexible experimental platforms at the centimeter scale with the primary goal of serving as a physical test bed for stochastic control strategies and corresponding modeling methods. Working at the centimeter scale facilitates developing novel experimental platforms (e.g., robotic modules, monitoring and agitation systems) and theoretical tools (e.g., modeling, control, optimization), which while being of considerable importance per se, represent a solid base for future downscaling and adaptation to platforms of much smaller dimensions. We believe that, in spite of the highly promising perspective of MEMS technology, long-range communication at sub-millimetric scales will only be feasible in the long term. As a result, relatively short-range interactions or physical contact shall remain the most viable means for achieving collaborative information sharing with the potential of being downscaled to smaller devices. Consequently, we consider physical aggregation a necessity to obtain collectively-coordinated responses from the system.

In the quest of a unifying framework for the modeling and control of SA of both active and passive modules, in this paper, we present the outcomes of our line of research during the past few years, involving three major studies. In the first study, we deploy an experimental platform comprising a water tank with peripheral pumps and a central station capable of controlling the pumps flows, as well as tracking the trajectories of several floating modules. Leveraging this experimental platform, we study the controlled SA of centimetric water-floating, magnetically-latching modules, not endowed with any sort of active control, called passive Lily modules. The formation of target structures out of passive modules is then modeled and controlled automatically in real-time and a closed loop using a dedicated multi-level computational framework [9]. This study validates the functionality of the interplay of the assembly and disassembly in distributed systems for efficient realization of SA. In the second study, using a similar experimental platform, albeit specific to a much smaller physical scale, we achieve controlled self-assembly of specially designed and fabricated microparticles of 200 μm into clusters of a preset dimension in a microfluidic chamber. The control loop is closed through real-time high-speed tracking and analysis of the system dynamics [10]. The third study comprises our latest results and focuses on distributing the intelligence and control over the SA process. It extends the centimeter scale experimental platform for the passive Lily modules to a flexible experimental system with the goal of studying fluid-mediated programmable self-assembly of active Lily modules. The versatile functionalities of the system allow for investigating a variety of control approaches, from fully centralized to fully distributed.

The rest of this paper is organized as follows. Section 2 provides an overview on the related works in the literature, categorized into two main classes: SA of miniaturized robots and SA of M/NEMS. In Section 3, the SA of passive Lily modules at the centimetric scale is explained, followed by a detailed description of its sub-millimetric counterpart, the SelfSys platform. Section 4 then presents the programmable SA of active Lily modules at the centimeter scale. A discussion on the outcomes of the work is presented in Section 5, where the paper also concludes, highlighting foreseeable future research.

## 2. Related Work

In this section, we provide an overview on the related works in the literature. We divide our review into two classes, the SA of miniaturized robots and the SA of M/NEMS.

### 2.1. Self-Assembly of Miniaturized Robots

The self-assembly of miniaturized robots has been studied in numerous works, where a wide range of hardware implementations, including fully-autonomous robots and controllable environments, along with corresponding control approaches, have been developed. These works differ mainly in the capabilities of the robots, i.e., the SA building blocks, the type of the environment and its level of controllability, and the approach employed to guide the SA process towards the target. The cubic modules presented in [11] are capable of forming structures in three dimensions deploying magnetic latching and a lattice-based locomotion approach on a test table. Self-assembly of a swarm of autonomous floating robots in two dimensions has been studied in [12]. In [7], programmable self-assembly has been demonstrated to be a powerful means for the formation of structured patterns in two dimensions in a large swarm of miniaturized robots, the Kilobots. While the motion of the Kilobots is inherently noisy, the primitive collective behavior programmed implements a deterministic and quasi-serial approach to shape formation. Taking advantage of the stochastic ambient dynamics for module transportation can, however, allow for the simplification of the internal design of the modules, as well as increased parallelization of the SA process. 3D stochastic SA of passive modules on an active substrate is investigated in [13]. Tribolon modules stochastically assemble into floating structures [14]. Tribolons are actuated using vibrating motors, and the environment is not capable of providing any control to guide the assembling process; the robots have a pantograph for both energy supply and control, including a latching mechanism based on the Peltier effect. The intelligent programmable parts in [15] are capable of local communication via infra-red and have controllable permanent magnet-based latches. The modules stochastically self-assemble on an air table, based on their internal behavior. The system of Pebble robots in [8] starts the process of shape formation with an ordered lattice; the stochastic forces in the environment are then used to detach unwanted blocks. Pebbles are only powered once they connect to the structure formed around the seed node; once connected and powered, they are capable of local communication among themselves. Abundant research has been dedicated to theoretical and experimental aspects of self-replication [16]. Self-replication of robotic units, for which self-assembled structure formation is crucial, is demonstrated using five 2D coding strings as templates [17] and also using self-swiveling microcontroller-based gripping blocks in [18]. Furthermore, a wide variety of novel platforms was developed in the context of modular robotics, some of which have the capabilities of autonomous locomotion and docking [12,19,20,21,22,23].

Both theoretical and experimental aspects of achieving SA and aggregation are of a high interest in distributed and modular robotics [20]. Probabilistic models of the SA of mobile robots are developed in several works [15,24,25,26]. Stochastic distributed control of robotic swarms is studied in [27], employing modeling methods originally developed for chemical systems [28]. In several other studies, the chemical formalism is demonstrated to suit the description of SA, both in real robot experimental scenarios and in simulation [9,29,30].

### 2.2. Self-Assembly of Micro-/Nano-Systems

Numerous applications are envisioned in the realm of SA of micro-/nano-systems, which demonstrate the potential and effectiveness of SA in providing alternative fabrication approaches across length scales and material interfaces. Landmark achievements include the SA of electrical networks [31], 3D electric circuits [32], integration of semiconductor devices into substrates [33,34,35,36], flexible LED surfaces [37], polyhedral containers [38] and monocrystalline solar cells [39]. Several types of physical interactions are employed: gravitational [40], hydrophobic [41], steric [42], electric [43], magnetic [44], capillary [45], DNA hybridization-mediated [46] and fluidic [47]. Most of these interactions have been shown to be tunable [48,49]. In almost all cases, the M/NEMS modules are designed, such that they are able to scavenge energy from the environment and exploit the information coded in physical templates. Miniature MEMS robots fabricated by micromachining capable of forming 2D structures several times their body size are demonstrated in [50]. Sub-millimetric MEMS robots utilizing a wireless resonant magnetic microactuator are demonstrated in [51]. An external AC electric field is used in [52] to control the electro-osmotic motion of light-responsive millimetric diodes. Self-folding miniature robots capable of being steered by an external magnetic field and dissolving into the environment are demonstrated in [53].

## 3. Fluidic Self-Assembly of Passive Modules

This section presents studies at two physical scales, first at the centimeter scale using the experimental platform around the passive Lily modules and then at the sub-millimetric scale using the SelfSys platform. The experimental platform at the centimeter scale shares several features with its sub-millimetric counterpart and, thus, serves as an emulator of the sub-millimetric case while providing more comfortable studying conditions at a scale where visual tracking and module fabrication are less challenging. Despite the substantial differences between the computational and control frameworks in the two studies, the control loop is closed using the feedback provided by the visual tracking of the modules in both cases.

### 3.1. Centimetric Scale

In this study, we address the stochastic SA of floating passive Lily modules [9]. In order to model and control the process, we employ a novel formal framework, denoted the $M^{3}$ framework. The framework has the capability to model and control the stochastic SA of fully passive modules in an automated and real-time fashion [9]. By tracking the modules’ trajectories, the framework automatically builds an internal representation of the system at the microscopic modeling level. This model then serves as a blueprint for models at higher abstraction levels. To calibrate these models, a Maximum Likelihood Estimation (MLE) approach is then employed.

#### 3.1.1. Module Design

**Figure 1:**
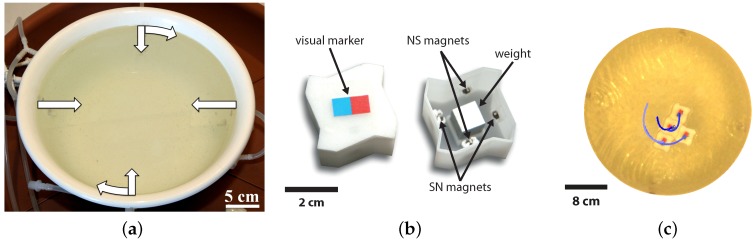
The experimental setup: (**a**) water-filled tank with six inlets (four orthogonal and two tangential to the wall); (**b**) details of a floating passive Lily module, including the latching mechanism composed of four permanent magnets with different pole orientation North-South (NS) and South-North (SN), respectively; (**c**) real-time visual tracking of four modules during an experiment (the blue lines show a short history of the trajectory of each module) .

A passive Lily module is a 3D printed plastic block of 3 cm in size endowed with four SmCo permanent magnets of 2 mm in diameter and 1 mm in height (one on each side’s center) as single-state latches. Each block is also endowed with a two-color visual marker for tracking purpose (see Figure 1b). The passive Lily module has a centro-symmetric rugged shape specifically designed for enhancing the alignment of interacting modules (Figure 1b). In order to improve flotation, an aluminum block is placed in the middle of the shell, reaching a total weight of 17.3 g, with the buoyancy limit being 21.9 g.

#### 3.1.2. Experimental System Design

**Figure 2:**
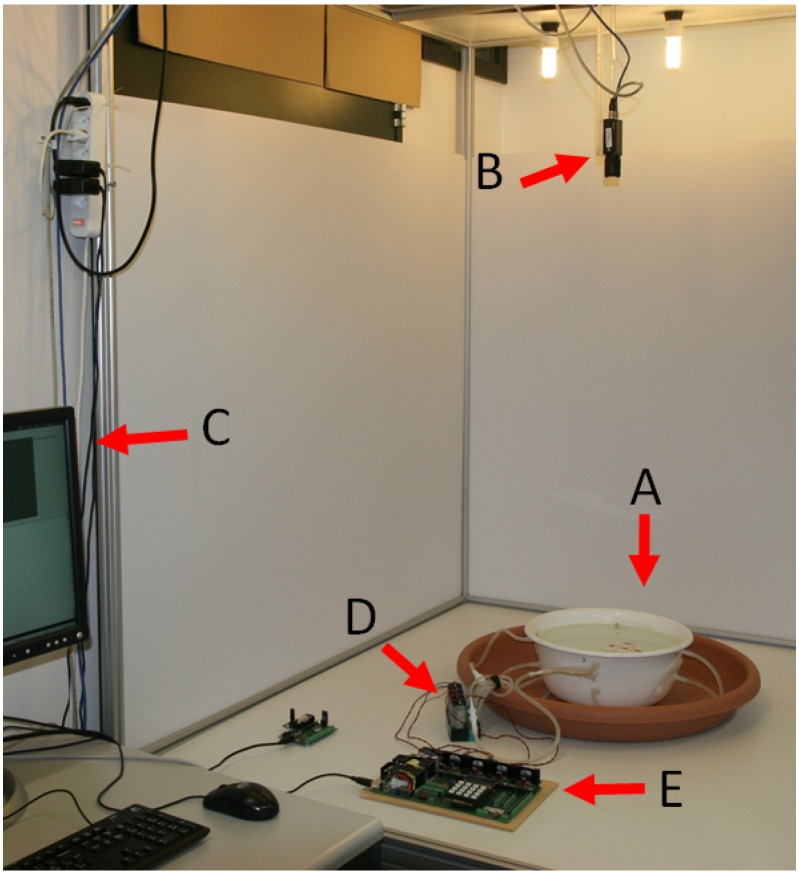
Experimental system, composed of water tank (**A**); overhead camera (**B**); base station desktop (**C**); diaphragm pumps agitating the fluidic environment (**D**); and control and driver board for pumps (**E**).

Figure 2 depicts the experimental system and its constituting components. Passive Lily modules are not self-locomoted; they are instead stirred by the fluid flow created by several peripheral pumps (Figure 1a). The fluidic arena includes a circular water tank of 30 cm in diameter, with four diaphragm pumps connected through six inlets. To create both radial and circular flows, four inlets are perpendicular and two are tangential to the wall. The four outlets are placed at the bottom of the tank to minimize perturbations to the surface flow. The flow rate of the pumps can be individually controlled up to 600 mL/min, allowing for a variety of flow patterns and, thus, a variety of module trajectories (Figure 1c). The perpendicular inlets create irregular trajectories, which favor collisions in the middle of the tank, but they tend to create dead spots along the walls. In contrast, the tangential inlets create regular closed circular flow patterns, which eliminate the dead spots along the walls. As a result, the combined flow patterns allow modules to exhibit trajectories with well-defined geometric features, but also with a strong stochastic component.

**Figure 3:**
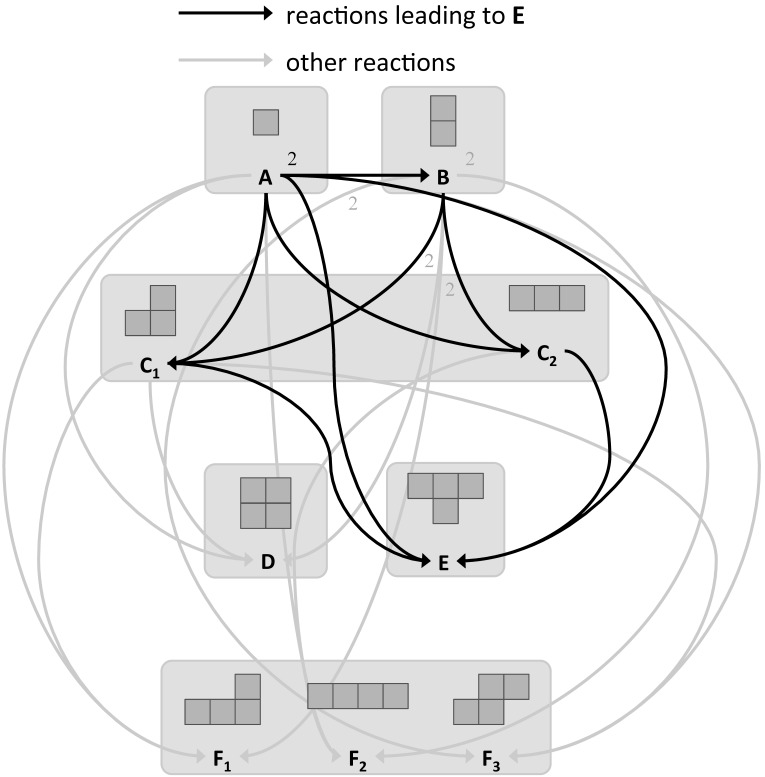
Graphical representation of all assemblies that can be formed out of four modules and the forward reactions giving rise to them. Chiral copies of assemblies $F_{1}$ and $F_{3}$ are not included. The shaded rectangles indicate assemblies with the same connection topology (using a four-neighbor topology). Black arrows denote the reactions that lead to the target structure E, whereas gray arrows other forward reactions in the system [9].

As a result of the interplay between the magnetic forces and the shape of modules, precise pair-wise self-alignment and latching is achieved between two modules at about a 0.5-cm proximity. The latching connection between modules is designed to be reversible. When connected, the magnitude of the magnetic forces between two modules is measured to be 16 mN per bond according to finite element method (FEM) simulations. Since the dipole approximation fails for close proximity, the magnetic force between two facing magnets was evaluated by FEM simulations as a function of relative distance. The reversibility of the latching connections was then experimentally fine tuned and validated by adjusting the placement of the magnets inside the Lily modules. With the latching connections being reversible, the stability of the assembled structures corresponding to local system energy minima can be influenced by changing the agitation mode in the tank. However, with four passive modules in the tank, the structure labeled D in Figure 3 corresponds to the global system energy minimum, meaning that a large amount of energy is required to break it up, resulting in an effectively irreversible structure. This state denotes an absorbing state in the system dynamics. In order to observe the evolution of the system in real time, an overhead camera is used to capture trajectories of the modules (see Figure 1c and Figure 2). The passive markers at the top of the modules are tracked using SwisTrack [54], an open-source software package developed in our laboratory. Using the two-color markers, the position and orientation of the modules are logged in real time at a rate of approximately 30 Hz and transmitted to the computational framework, which handles the online construction of a model of the system and also the optimization of the agitation as described in the following section.

#### 3.1.3. Modeling and Control

We consider the problem of controlling the SA process in a system consisting of four passive Lily modules towards constructing a target structure. More formally, the research question we would like to address is the following: given a stochastic multi-unit system with a finite set of control inputs, i.e., agitation modes, $M=\{m_{0},\ldots ,m_{n}\}$, what is the mode $m_{i}$ to be selected at time *t* considering the current population state of the system $x_{t}$ in order to minimize the time to form a given target structure *T*?

Here, we consider only two agitation modes (i.e., a bang-bang controller). At mode $m_{0}$, the flow pattern gives rise to smooth and regular module trajectories with small differences in their relative velocities. As a result, this mode allows for a high stability of the formed structures, but relatively few collisions. At mode $m_{1}$, the flow pattern gives rise to much more erratic trajectories, which are mostly dominated by the stochastic perturbations of the water surface (Faraday waves) caused by the pump-induced vibrations of the tank. As a result of the higher kinetic energy of the modules, the collision rate and the instability of the formed structures increase. All feasible structures formed out of four Lily modules can be reached in our setup (see Figure 3). Our methodology makes a fundamental assumption: the modules are strictly reactive, meaning that all behavioral changes can be ascribed to interactions with other modules or the environment. As a result, the interaction configuration of the modules fully describes their behavior.

**Figure 4:**
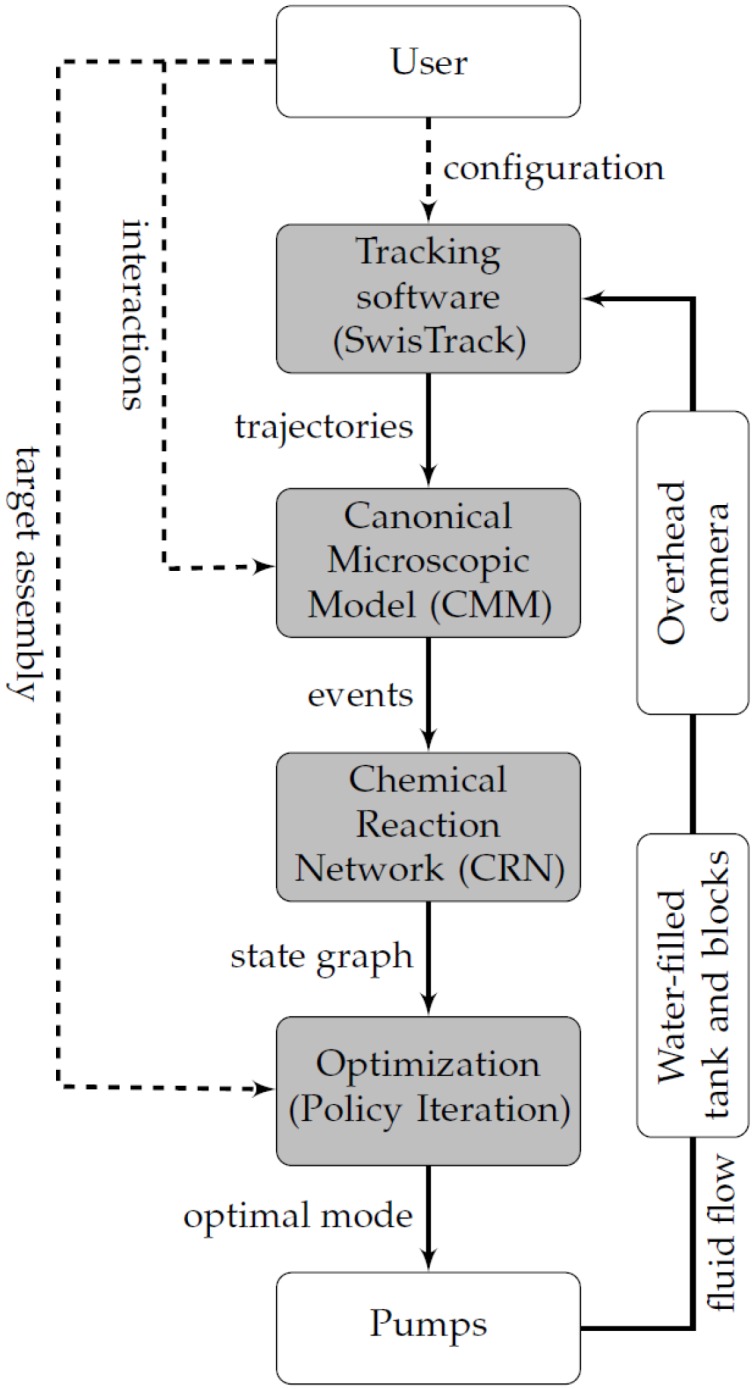
Overview of the $M^{3}$ framework as deployed in this study and the different types of information flowing between its constitutive modules [9]. Gray-shaded nodes are computational entities, whereas other nodes are physical entities. Dashed arrows denote flows that are not automated, but need to be performed only once prior to the experiment. Note that the closed-loop control is completely automated.

Following the aforementioned arguments, the $M^{3}$ computational framework is developed, which allows for automatically constructing models of our multi-unit system. The overall structure of the $M^{3}$ framework is depicted in Figure 4. The framework builds a microscopic-level representation of the system based on a set of user-specified interactions and also observations of the trajectories of the units. This representation, denoted the Canonical Microscopic Model (CMM), captures the system as a set of coupled hybrid automata [55], which interact through a set of interactions. The CMM provides a formal and generic description of a reactive multi-unit system and serves as a blueprint for the construction of a macroscopic model, specified using the Chemical Reaction Network (CRN) formalism. Using an optimization scheme, the optimal agitation mode is then determined at each time step and applied to the real or simulated actuators.

The optimization problem is equivalent to solving the Markov Decision Process (MDP) model of the system. Assembling the target structure *T* is equivalent to reaching a target population ${\vec{x}}_{t}=\;\left(x_{t,1},\ldots ,x_{t,M}\right)$ where:

(1) $$x_{t,i}=\left\{\begin{array}{cc} 1 & \text{if}\;S_{i}=T,\\ 0 & \text{otherwise}. \end{array}\right.$$

The optimization problem is thus to choose the agitation mode $m_{s}\in M$ to be applied given an initial population ${\vec{x}}_{s}$, such that the expected time to reach ${\vec{x}}_{t}$ is minimized.

For each mode m∈M, the CRN model contains an estimate of the propensity function $a_{R}^{\left(m\right)}\left(\vec{x}\right)$ for each reaction R∈R. Assume $k_{ij}^{\left(m\right)}=a_{R}^{\left(m\right)}\left({\vec{x}}_{i}\right)$ to be the rate of the reaction *R* with an associated population change of ${\vec{\nu }}_{R}={\vec{x}}_{j}-{\vec{x}}_{i}$ given that mode *m* is selected. Furthermore, assume the following:

(2) $$\begin{array}{ccc} \lambda_{i}^{\left(m\right)}=\sum_{j}k_{ij}^{\left(m\right)}, & & p_{ij}^{\left(m\right)}=\frac{k_{ij}^{\left(m\right)}}{\lambda_{i}^{\left(m\right)}}. \end{array}$$

Assume $T_{ij}$ to be the optimal first-passage time from population ${\vec{x}}_{i}$ to ${\vec{x}}_{j}$. In order for $T_{ij}$ to be optimal, it can be shown that the following should hold true:

(3) $$T_{it}=\min_{m\in M}\left\{\sum_{j\neq i,t}p_{ij}^{\left(m\right)}\cdot T_{jt}+\frac{1}{\lambda_{i}^{\left(m\right)}}\right\}$$

This equation is the Bellman equation corresponding to the MDP, which models the system and can be solved to obtain the expected time and the optimal agitation mode for a given initial population state. We employ the policy iteration method to solve these equations. The optimization is performed in an event-based fashion, upon each assembly or disassembly event.

#### 3.1.4. Results and Discussion

Four different experimental scenarios are investigated with the target assembly E depicted in Figure 3, in order to evaluate our automatic model building framework. Each scenario employs a different control method: (I) only agitation mode $m_{0}$; (II) only agitation mode $m_{1}$; (III) random agitation mode control, where the two agitation modes are applied randomly with an average switching period of 15 s; and (IV) optimized control, where the appropriate agitation mode is selected by the optimizer according to the current state of the system and the model. The performance is evaluated by the first occurrence of the target assembly E, i.e., the first-passage time, starting from the initial state of fully-isolated modules scattered randomly in the arena. Each experiment is 30 min long and is repeated 40 times. As the experiments progress and more observations are made, the model gets constantly enhanced. However, in Experiment IV, an initial model is provided to the optimizer. This initial model is built by making observations in two sets of experiments (one per mode) of 10 runs, each 5 min long.

**Figure 5:**
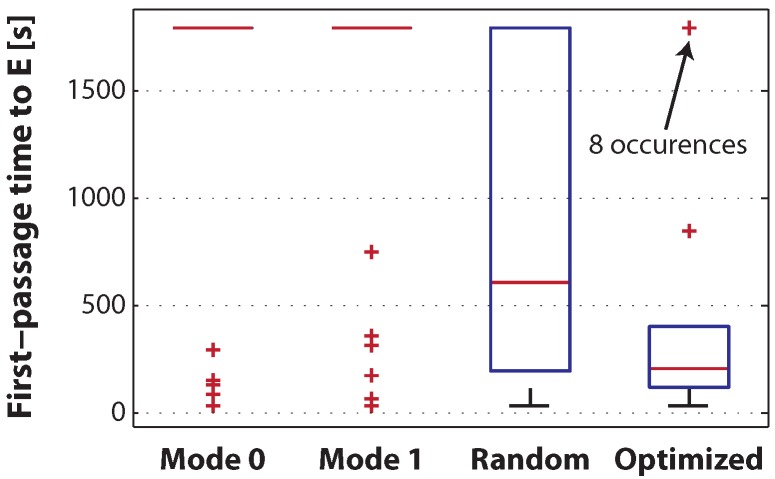
Box plot of the first-passage time to the target structure E obtained over 40 runs of 30 min each for Experiments I to IV. On each box, the central mark is the median; the edges of the boxes are the first and third quartile; the whiskers extend to the most extreme data points not considered outliers; and outliers are plotted individually. Both Experiments I (Mode 0 only) and II (Mode 1 only) exhibit a poor performance due to the unfavorable exploration vs. exploitation balance when using a unique mode of agitation. The mean/median first-passage time of the optimized experiment (IV) is 524/205 s versus 930/612 s for the randomized experiment (III). A Mann–Whitney test rejects the null hypothesis of the two distributions of first-passage times being from the same distribution with equal medians with a *p*-value of $5.8\times 10^{-3}$ [9].

The experimental results are depicted in Figure 5. It can be seen that both control Methods I and II perform poorly even compared to the fully-random strategy. This can be ascribed to the fact that self-assembly, like other self-organized phenomena, requires a subtle trade-off between “exploitation” and “exploration”, here equivalent to the low-agitation $m_{0}$ and the high-agitation $m_{1}$ modes, respectively. The optimized controller on the other hand exhibits a 40% and 66% decrease on the average and median of first-passage time, respectively. The optimized strategy can also be interpreted intuitively: the low agitation mode $m_{0}$ is active as long as assemblies that may lead to E (i.e., assemblies A, B, $C_{1}$ and $C_{2}$) form. When an incorrect tetramer is formed, the strong agitation mode $m_{1}$ is activated. In addition, several non-intuitive behaviors are also observed. First, when the system comprises solely isolated modules, the agitation mode $m_{1}$ is activated favoring mutual collisions. Once a dimer B is formed, the system may activate the agitation mode $m_{0}$ in order to preserve it. However, it is interesting to note that while most reactions rates are significantly different for the agitation modes $m_{0}$ and $m_{1}$, the reaction $A+B\to C_{x}$ has relatively similar rates for both modes. As a consequence, an interesting adaptive behavior is observed. The optimizer might initially select the low agitation mode $m_{0}$ to preserve the formed dimer. As the experiment progresses, the model gets updated, and the reaction rate of trimer creation in mode $m_{0}$ decreases until it falls below that of the mode $m_{1}$, resulting in activation of the agitation mode $m_{1}$. This adaptive behavior is an inherent feature of our automated on-line modeling approach.

### 3.2. Sub-Millimetric Scale

**Figure 6:**
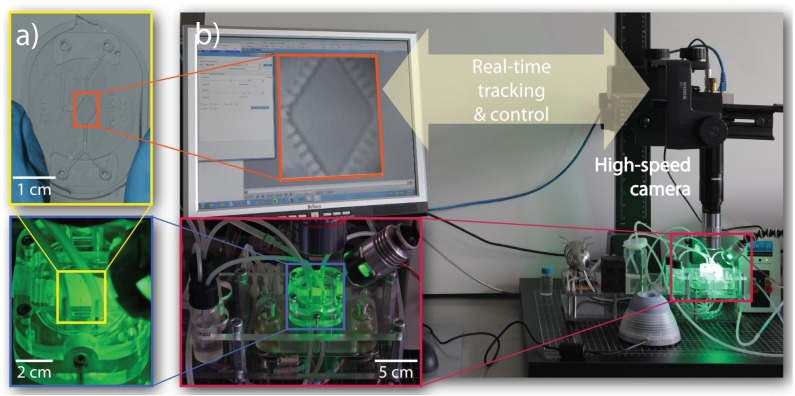
The automated, closed control loop of the SelfSys platform [10]. (**a**) The 1 cm long, centro-symmetric chamber molded in a 400 μm-thick layer of polydimethylsiloxane (PDMS) and sealed between two glass slides; (**b**) The high-speed GigE camera (Fastec HighSpec 1) with a microscope lens assembly mounted above the chamber for tracking purpose.

Our previous study with the passive Lily modules at the centimeter scale provided evidence on the functionality of the interplay between assembly and disassembly events towards efficient SA in distributed systems where reversible bonds are formed between the agents. Self-assembling systems at the microscopic scale typically employ open-loop and non-automated methods for controlling the position and motion of microagents [56,57]. Here, we present SelfSys [10], a novel experimental platform capable of controlled fluidic SA of microparticles (Figure 6). Sharing several features with the passive Lily modules platform, the SelfSys platform comprises a water-filled microfluidic chamber where the SA process is driven by the agitation modes induced by a coupled ultrasonic actuator. The SA building blocks in this platform are the three-dimensional polymeric microparticles, which, similar to the passive Lily modules, scavenge their motion from the acousto-fluidic flow field. A dedicated software framework is developed, which is capable of imaging, tracking and analyzing in real-time the fast dynamics of the microparticles, as well as switching the agitation modes in the microchamber accordingly. The SA process in the SelfSys platform employs a fully-automated closed control loop in order to direct the stochastic SA process towards achieving a target cluster of a preset dimension as opposed to a target with a specific shape, as in the case of the passive Lily modules discussed in Section 3.1.

#### 3.2.1. Module Design

**Figure 7:**
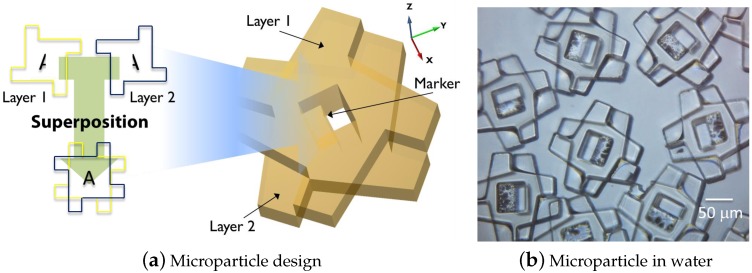
(**a**) 3D design of the microparticles for fluidic self-assembly (SA); (**b**) microfabricated SU-8 microparticles in water [10].

Figure 7 depicts the three-dimensional polymeric microparticles fabricated as the building blocks of the SA process in the SelfSys platform [58]. The profile of these microparticles is similar to that of the passive Lily modules [9] (Figure 7a), facilitating self-alignment of the interacting particles into a closely-packed lattice. The microparticles (Figure 7b) were fabricated through the deposition and patterning of two 50 μm-thick layers of SU-8 using thick electroplated copper as the sacrificial layer [58]. In order to increase the hydrophobicity of the SU-8 surfaces, they were coated with a fluorinated silane-based self-assembled monolayer. When placed in close proximity, the surfaces of the microparticles interact through short-ranged hydrophobic effects [59]. The drag forces created by the liquid can overcome the inter-particle hydrophobic effects, as well as the secondary radiation forces created by the ultrasonic field, allowing the bonding between the microparticles to be reversible. In order to facilitate the visual tracking of the microparticles, they feature a bichromatic marker in the middle created by a simple design of a pair of steps.

#### 3.2.2. Experimental System Design

A 1 cm-long centro-symmetric water-filled microfluidic chamber hosts the SA of the microparticles and is at the center of the SelfSys platform [60] (Figure 6a). The chamber is molded in a polydimethylsiloxane (PDMS) layer of 400 μm in thickness sealed between two glass slides (Figure 6a). The water flow is controlled using a pump. The microchamber has an acoustic resonance frequency of 40 kHz and is in contact with an ultrasonic piezoelectric actuator with a resonance frequency of 60 kHz. The PDMS side is surrounded by an array of 20 air-filled, 500 μm-long sockets. With the air sockets being arranged in a symmetrical placement, a central laminar flow is generated, which drags the immersed microparticles. The streaming flow generated by the oscillating bubbles induces efficient mixing in the chamber in spite of the chamber’s low Reynolds number (*Re* ranges from 12 to 112 depending on the actuation frequency; for more details, see [60]).

Similar to the case of the centimeter-size passive Lily platform [9], two agitation modes of assembly and disassembly are employed by tuning the actuation frequency of the piezoelectric actuator to the resonance frequency of either the air bubbles trapped in the peripheral sockets or that of the chamber, at maximum amplitude. At the assembly agitation mode, the microparticles are driven to aggregate in the middle of the chamber. At the disassembly agitation mode, the formed clusters are driven to dismantle in the proximity of the walls.

**Figure 8:**
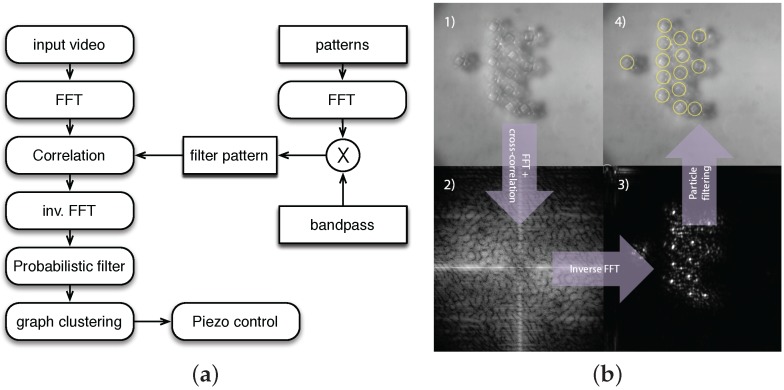
(**a**) Diagram of the particle tracking system in the SelfSys. The generation of the filter pattern is run only once, thus it does not contribute to the total processing time during operation [10]; (**b**) Image processing steps in the SelfSys: (**1**) captured input image; (**2**) bandpass-filtered cross-correlation (Fourier domain); (**3**) matching result after inverse Fourier transform and probabilistic filtering; (**4**) final tracking displayed on the input image [10].

To track the trajectories of the microparticles, a dedicated high-speed imaging solution was implemented. The full tracking software structure is represented in Figure 8a. The open source software package SwisTrack [54] was used again in this case study, but augmented with new modules capable of pattern-based particle tracking. Several challenges had to be dealt with, including the blur due to the small field depth and also the varying low contrast of the transparent floating microparticles against the background. We chose an approach based on the cross-correlation of the pattern of a microparticle against a captured image in the Fourier domain. The captured image goes through a 2D fast Fourier transform (FFT) unit; it is then multiplied with the 2D-FFT of a microparticle pattern. In order to cope with the conditions of varying blur and lighting, the microparticle pattern initially goes through a bandpass filter. This allows one to cope with the conditions of ambient lighting variations (i.e., low frequencies) and also image noise and specular reflections (i.e., high frequencies). The cross-correlation amplitudes are then passed through an inverse FFT block and searched for maxima corresponding to the best matching with the original input pattern. In order to reduce the false positives and improve the performance of the tracking, the result of the inverse FFT block goes through a Bayesian filtering block, which produces a new estimate by combining the current observation with a Gaussian probability distribution of previous matches. The described image processing algorithm is partially implemented on a Graphics Processing Unit (GPU), thus allowing for the tracking system to run at the full capture rate of the camera at 150 Hz. A block diagram of the algorithm is shown in Figure 8b.

#### 3.2.3. Modeling and Control

Similar to the study with passive Lily modules, here our objective was to direct the stochastic SA process towards achieving clusters of microparticles with a user-defined target size. The position of the microparticles in the camera field of view is provided instantaneously by the tracking algorithm. According to the current system state, an actuation mode of assembly or disassembly is automatically chosen such that the system progresses towards forming the target cluster at each step. Based on the output image of the microparticle tracker, a graph G(V,E) is constructed encoding the configuration of the current cluster, with *V* being the set of identified particles in the cluster and *E* being the set of physical links between the particles. At each time instant, the size of the current cluster $c_{t}$ and the target cluster size *γ* are compared. When $c_{t}$ is smaller or larger than *γ*, the actuation mode is set to assembly or disassembly mode by setting the piezoelectric actuator, respectively. Once at least one cluster with the target size is present in the system (i.e., when $c_{t}=\gamma$), the actuation is immediately stopped. As a result of the low Re of the microchamber, the motion of the microparticles also stops almost instantaneously following the stopping of the actuation.

**Figure 9:**
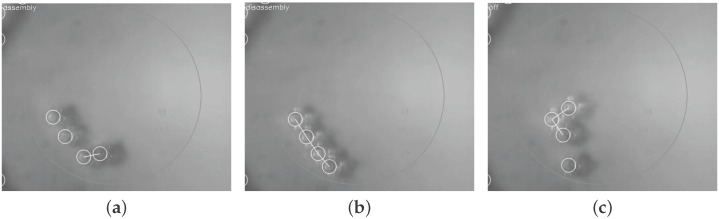
Self-assembly sequence for target cluster of size: three [10]. (**a**) 2.00 s, assembly mode; (**b**) 2.80 s, disassembly mode; (**c**) 3.92 s, target structure achieved.

**Figure 10:**
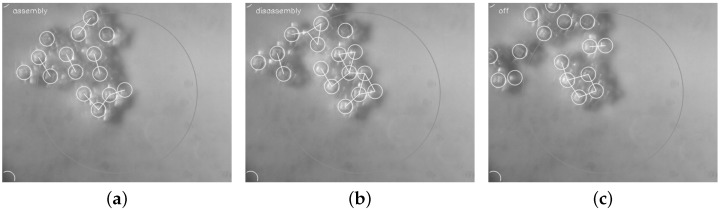
Self-assembly sequence for a large microparticle set. Dimensions of the target cluster: five [10]. (**a**) 7.04 s, assembly mode; (**b**) 7.24 s, disassembly mode; (**c**) 7.68 s, target structure achieved.

By combining the described tracking and also the control software components, an automated closed-loop control strategy is achieved for the SelfSys platform [10]. More specifically, the platform achieves a fully automated evolution of the system from an initial state consisting of isolated microparticles to one with connected clusters of the target size in real time in spite of the inherently fast and highly stochastic system dynamics. Figure 9 and Figure 10 show snapshots from two realizations.

Numerous experiments on the SA process validate our control approach [10]. Several points should be highlighted here. With the PDMS sidewalls being out of the camera field of view, the microparticles stuck to the sidewalls cannot be tracked by the developed software framework. Consequently, with the microparticles being able to enter and leave the camera field of view, the number of tracked microparticles is typically not constant over time. This results in the system state to be only partially observable in contrast to its centimeter-size counterpart [9]. Two major consequences thus follow: (1) accumulating statistics on the system performance becomes more challenging; and (2) control strategies based on CRN modeling and expectation-maximization algorithms as in the centimeter-size counterpart [9] are not applicable here. The second consequence was in fact a key motivation for the simple bang-bang control method employed, which was able to cope with the partial observability condition.

## 4. Fluidic Self-Assembly of Active Modules

**Figure 11:**
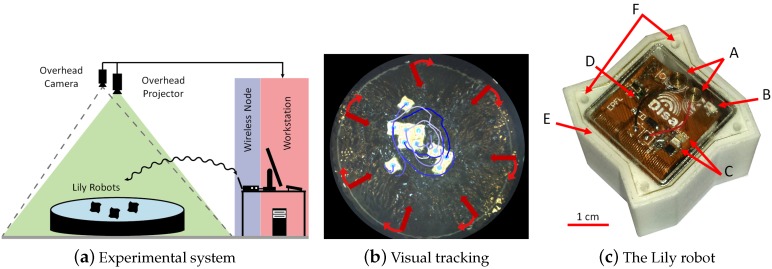
(**a**) The experimental setup consisting of a water-filled tank with peripheral pumps agitating the fluidic environment, an overhead camera and a projector, a wireless node for establishing the radio link between the workstation and the Lily robots; (**b**) Visual tracking of ten Lily robots during an experiment (the blue lines show a short trajectory history for each robot); (**c**) The Lily robot [61]; some key features visible in the picture are: charging contacts (A), chip antenna (B), two LEDs signaling board status (C), ambient light sensor (D), sealing gap filled with silicone paste (E) and two of the four trimming holes (F).

In this study, we extend the design of the passive Lily modules to achieve programmable devices, which we will call interchangeably active modules or robots. We then study fluid-mediated programmable stochastic self-assembly of robots and present a full experimental system that allows for exploring the whole range of fully-centralized to fully-distributed control strategies for programmable stochastic self-assembly of 2D structures on water, as depicted in Figure 11. The system is built around the water-floating 3-cm-size Lily robot and is an extension over the experimental system used with the passive Lily modules introduced in Section 3.1.

### 4.1. Module Design

**Figure 12:**
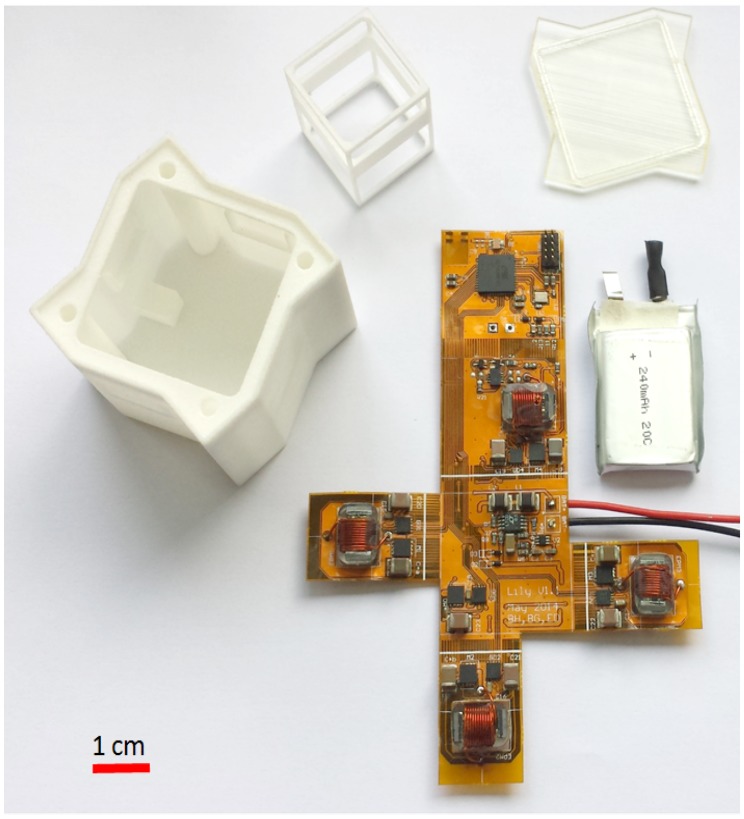
The Lily robot comprises a flexible circuit board with four Electro-Permanent Magnets (EPMs) soldered on it, a 240 mAh LiPo battery, a 3D printed shell, a 3D printed transparent cap and a 3D printed frame for holding the EPMs in place [61].

Figure 11c shows the intelligent Lily robot, a 35-mm cubic-shaped water-floating power-autonomous robot. As can be seen in Figure 12, each robot comprises several 3D printed plastic parts, a LiPo battery, four Electro-Permanent Magnets (EPMs) as the latching connection and local communication mechanisms and a flexible Printed Circuit Board (PCB). The PCB is two-layered with a total of 230 μm in thickness, hosting a microcontroller unit with an integrated radio transceiver, analog radio front-end circuitry and a switching and power circuitry. The EPMs are soldered on the flexible PCB, which is then folded and put on top of the battery sitting at the bottom of the plastic shell, with the EPMs snapping into dedicated sockets. In order to fine-tune the weight distribution and allow for a balanced flotation, four trimming cavities are devised in the shell (Figure 11c. The 3D printed shell inherits its specific rugged shape from the passive Lily modules described in Section 3.1.

**Figure 13:**
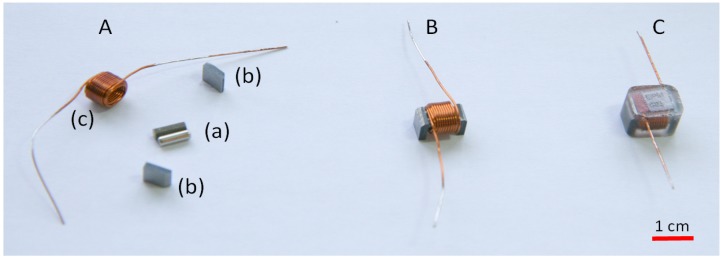
(**A**) An EPM is composed of two magnet rods of different coercivities, but similar remnant fields (a), sandwiched between two iron pole pieces (b), and wrapped with 32 turns of grade 26 American wire gauge (AWG) wire (c); (**B**) The pieces are hold together using glue; (**C**) The assembly is then put in a polyurethane mold for protection against rusting [61].

The choice of the latching mechanism was driven by several motivations. On the other hand, automatic alignment of the latching faces is a crucial feature due to the inherently stochastic and high energy interactions in the system. On the other hand, long-term energy autonomy and buoyancy requirements prompted for a low-power and small-size latching mechanism. EPMs meet such requirements and offer the following advantages: they consume power only during the transient switching time; they can be efficiently downscaled (the required switching energy being proportional to the volume, and the effective force being proportional to the area of the latch [62]); automatic alignment of the latches can be naturally obtained as a result of the interaction of the EPMs magnetic fields. Figure 13 shows a custom-sized EPM employed in the Lily robots. An EPM consists of two magnet rods wrapped in a coil and sandwiched between two iron poles to direct the magnetic flux. The two magnets are of different types, both having almost the same remnant magnetization, but very different coercivities, i.e., one being a soft and the other being a hard magnet. A sufficiently strong peak of current through the coil switches the polarization of the soft magnet aligned or opposite to that of the hard one and thus switches the latch “on” or “off”, respectively. The current pulse is obtained by discharging a capacitor on the EPM coil. At the state “on”, magnetic materials are attracted by the magnetic flux reaching out through the iron poles; at the state “off”, the magnetic flux flows through a closed low resistance path provided by the iron poles.

Finally, because it can be impractical and tedious to program, turn on or off, or charge a large number of robots individually, the Lilies are specifically designed in order to minimize individual handling for the aforementioned operations, as well as starting, pausing or stopping an experiment.

### 4.2. Experimental System Design

The experimental setup is an extension over the setup used with the passive Lily modules and consists of a circular water-filled tank equipped with peripheral pumps, an overhead camera, an overhead lighting system comprising a projector and an LED lamp, a wireless node communicating with the robots and a workstation (see Figure 11). The tank is approximately 1.2 m in diameter and 0.3 m in depth and has seven inlets perpendicular to the wall, which are endowed with a small insert piece to deviate the flow by about 15 degrees, creating a flow field with both radial and circular components. The flow rate of each pump can be individually controlled up to 9 L/min, allowing for a wide variety of fluidic fields and induced trajectories. To follow the evolution of the system, we use an overhead camera to monitor a passive marker located at the top of each robot using again SwisTrack as the tracking software [54]. The positions of the markers are logged at approximately 30 Hz. Complementary to the visual tracking data are the data logged by the wireless node communicating with the robots over radio. These data contain the evolution of the robots’ internal states. The wireless node is also used to program the robots and send commands or feedback to them during the experiment, potentially enabling the robots to adapt their behavior according to a global image of the SA process, rather than the robots’ local perception.

### 4.3. Modeling and Control

Structure formation through SA by a swarm of Lily robots comprises several aspects. Given a target structure, a proper behavioral rule set is derived using our dedicated framework [63] and deployed on all robots through wireless bootloading. The EPMs in each robot are enabled by default. Once two robots are connected, the inductive EPM-to-EPM communication channel is established physically. The robots then communicate their internal states and based on their behavioral rule set, they will either decide to unlatch or remain latched and update their internal states accordingly. The robots then update the central station computer with their new internal state through the wireless link. In order to reject an interaction, the robots both disable their engaged EPMs for a certain time during which the environmental fluidic agitation will drift them apart. In addition to communicating their internal state in an event-based fashion, the robots constantly communicate with the central station computer checking for possible pending commands, such as a battery level query, a pause or a stop command. This scheme allows for extending the battery life by keeping the radio off most of the times. The central station computer commands may also be used to update the robots’ rule set behavior on the fly.

We employ an extended version of the graph grammars formalism to formulate the problem of rule synthesis for SA of robots [63]. Standard graph grammars formalism has been previously used for the modeling and control of the SA of abstract graphs [15,64]. The standard formalism models a self-assembling system as a graph with the vertices corresponding to the building blocks and the edges of the graph corresponding to the bonds between the building blocks. Given a desired target graph, a rule synthesis algorithm would then synthesize behavioral rule sets to build the target. The limitation with using the standard formalism for describing the SA of robots is that the morphology of the latching connectors of the robotic modules cannot be readily incorporated into the graph. One solution has been to represent each latching connector by one node in the graph of the system and connect them with permanent links to represent one robotic module [15]. Within the extended formalism, the system is represented as an extended graph with extended vertices. An extended vertex has a set of ordered link slots, which correspond to the latching connectors of the robotic modules, thus directly incorporating the morphology of the robotic modules in the graph structure. We have shown that for robotic modules with *N* connectors, the extended graph grammar formalism can lead to rule sets with O(N) complexity as opposed to the $O(N^{2})$ resulting from the standard formalism [63]. In the same work, we show how formal rule synthesis algorithms proposed for SA graphs can be extended to synthesize rules for the SA of robotic modules using the extended graph grammar formalism. In this work, we employ rule sets generated by the extended Singleton algorithm as presented in [63].

In order to characterize the system and validate its functionalities, we conducted multiple experiments; here, we highlight relevant sets. In Experimental Set A, the purpose is to assess the applicability of the standard chemical kinetics modeling to our system and, thus, to show the relevance of our previous modeling efforts, discussed in Section 3.1, to the Lily robots and their related setup [9]. Experimental Set B is intended to demonstrate and validate the capabilities of the system to accomplish the self-assembly of several structures, also while adapting the behavior of the robots on the fly, for instance according to the perceived ambient luminosity or specific radio messages, possibly emulating environmental clues.

### 4.4. Results and Discussion

All of the experiments are conducted using 10 Lily robots, described originally in [61], and using three main flow regimes, referred to as high, medium and low agitation modes hereafter, corresponding to 6-L/min, 4.4-L/min and 2.6-L/min flow rates for all pumps, respectively. For gathering statistics, the experiments were run 10 times. The error bars/envelopes denote one standard deviation interval around the mean value.

#### 4.4.1. Experimental Set A: Macroscopic Kinetics

**Figure 14:**
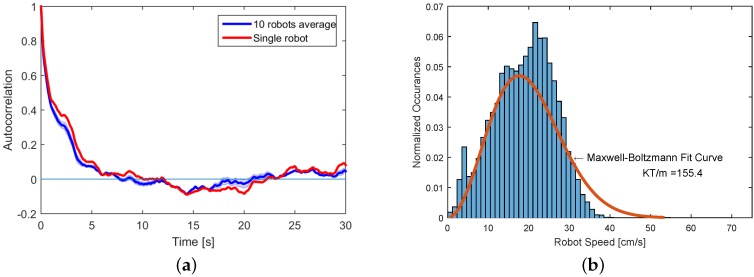
Macroscopic kinetics of the system. (**a**) Autocorrelation of the speed (i.e., magnitude of velocity) for Lily robots. The speed is uncorrelated to its initial value after approximately 5 s. The shaded area signifies one standard deviation interval; (**b**) Distribution of the robots’ speeds acquired based on the tracking data with normalized occurrences (total sum of one).

**Figure 15:**
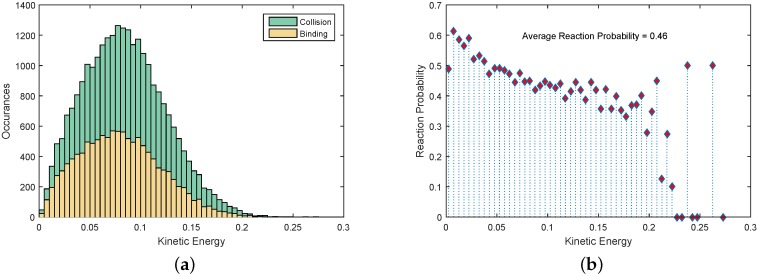
Reactions in the system. (**a**) Distribution of collision and binding events across the collision energy spectrum; (**b**) reaction probability distribution across the collision energy spectrum.

Our previous work proposed a computational framework for automated modeling and control of SA processes of a small number of passive modules [9]. The assumption of the framework was that the system was governed by reaction-diffusion dynamics. This allowed us to apply canonical chemical kinetics models. In this experimental set, we investigate the validity of such an assumption for the system of active Lily modules. Diffusing particles exhibit Brownian motion with a Maxwell–Boltzmann velocity distribution. A characteristic of such particles is that they quickly forget their velocities [15,65]. For this experimental set, the robots were programmed with an empty rule set, thus immediately detaching upon binding events. Figure 14a shows the autocorrelation of the speed (i.e., magnitude of velocity). The speed of each robot after approximately 5 s is effectively uncorrelated with its initial value. These observations support the assumption that the Lily robots approximately undergo diffusion while being stirred by the flow field across the fluidic arena. Additionally, as a side result, we qualitatively compare the speed distribution of robots with a Maxwell–Boltzmann distribution. The distribution of the robots’ speeds in a 30-min experiment using the high agitation mode is shown to be approximately of the Maxwell–Boltzmann type, as depicted in Figure 14b. Further experiments and statistical tests should be carried out for each experimental scenario, characterized by a possibly different agitation mode or density of robots, to generally validate this condition. Figure 15a depicts the distribution of collision and binding events across the collision energy spectrum. The horizontal axis is the sum of the kinetic energies of the colliding robots per unit mass, computed based on the data gathered by the tracking system. It can be seen that while most of the collisions have an energy content around 0.075 m^2^/s^2^ per unit mass, the binding probability is higher for collisions of lower energies. This might be better observed in Figure 15b, where the reaction probability, i.e., the ratio of binding over collision events, is shown across the kinetic energy spectrum. The average reaction probability for the high agitation mode is measured to be 0.46.

To add the notion of time, we can compute the stochastic reaction rate by combining the diffusion rate, which defines the time span between collision events, and the reaction probability, which defines the probability of a collision event resulting in a bond [66]. It is also possible to theoretically obtain the stochastic reaction rate based on the geometry and dynamics of the system [67]. These rates can then be used to calibrate a highly abstracted and non-spatial model of the system. We plan to report on this front in a future work.

#### 4.4.2. Experimental Set B: Demonstrating Lily Robots’ Capabilities

For the following set of experiments, the robots were programmed with appropriate rule sets derived using a dedicated framework [63]. In addition to the static rule sets, the robots constantly read the light or radio message values, based on which they choose to pick certain rules in their rule set.

**Figure 16:**
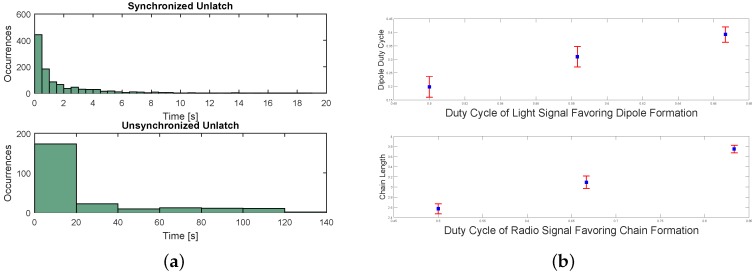
(**a**) Interaction time distribution: synchronized unlatching can be seen to allow for more efficient breaking of a bond; (**b**) dynamic behavior changes upon perceiving environmental light.

In the first experiment, we study how the coordination between two robots turning their latched EPMs off affects the unlatching interaction. The robots were programmed with an empty rule set and two cases were studied. In the first case, the robots perform their normal unlatch behavior, turning off their EPMs synchronously, while in the second case, only the robot that has initiated the communication turns off its EPM. As can be seen in Figure 16a, the interaction time distribution tends towards significantly larger values for the second case, suggesting that the robots have more difficulty to detach. These results highlight the crucial role of the handshaking process between the two neighboring robots involved in an unlatched dynamic. The handshaking process is crucial to reliably ensure that the robots are mutually aware of their the decision and timing for turning their EPMs off.

In the second experiment, the fluidic arena is lit up using the overhead lamp. The Lily robots perceive the green light using their on board light sensor. When the light is off, the environmental condition is considered to be undesirable for assembly. When the light is on, the condition is in favor of dipole formation. The light signal changes periodically, with each experiment having a different duty cycle of the light being on. In a real-world application, this scenario may be associated with a medication delivery application where the particles enclose some medication while forming a dipole and release the substance only when they are located in the target region defined by a certain desirable condition. Additionally, in a similar scenario, the Lily robots periodically receive a message over the radio signaling them to switch their behavior from dipole formation to the formation of chains of unrestricted lengths and the other way around. We change the duty cycle of the message and measure the average length of assemblies in the system. Figure 16b depicts the dipole life time versus the duty cycle of the light signal, as well as the average length of the chains versus the radio signal duty cycle. In Figure 16b top, it can be seen that the time that the robots spend in a dipole formation increases accordingly with the duty cycle of the light signal, meaning that the system successfully detects the environmental condition and behaves accordingly. Figure 16b, bottom, shows that the average length of the chains increases accordingly with the signal duty cycle, meaning that the system successfully behaves according to the feedback from the central node.

**Figure 17:**
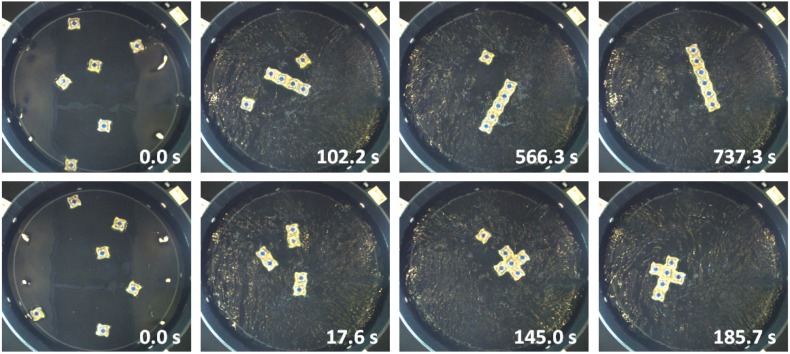
The progress of the SA process for the chain and cross shape target structures.

In the third experiment, we programmed six Lily robots with rule sets synthesized by our framework to build serially two target structures (chain and cross shapes) [63]. Using rule sets that are executed by the individual robots allow us to follow a fully-distributed control approach to guide the SA process. Each experiment was repeated five times. For all cases, the formation of the target structure was achieved within 13 min with the fluidic flow field being fixed at the high agitation mode. The robots would collide, communicate their internal state, browse their rule set for an applicable rule, remain attached and update their internal state upon finding one. In the case that no applicable rule was found, i.e., the robots participate in a non-desirable sub-assembly, the robots would use a synchronized latching maneuver to detach. Figure 17 shows the progression of the SA process of one run for each specific target structure.

## 5. Discussion and Conclusions

In this work, we presented a series of experimental and theoretical developments, building structures through fluid-mediated stochastic self-assembly. Our research addresses the design, modeling and control aspects of self-assembly in engineered systems. One major question in engineered self-assembling systems is how the SA process can be induced or guided, such that it culminates in specific desired target structures while at the same time maximally leveraging the intrinsic concurrent building of SA. Depending on the capabilities of the building blocks and the controllability of the environment, a wide variety of control approaches, from centralized to distributed, may be employed to guide the process. In particular, we presented three main studies, with each study contributing to both our theoretical and technological research thrusts as outlined before, as well as setting a solid base for the subsequent ones.

We exploited several common tools and methods for these studies. In particular, in each study, we carefully designed and developed the interaction means and hardware characteristics of the modules, depending on the feasible technology and module complexity at the corresponding scale. In all studies, we carried out SA experiments in fluidic environments where the modules were not self-locomoted; instead, they scavenged their motion from different agitation modes induced in the fluidic arena. Additionally, we monitored the evolution of the SA process in the system using visual tracking, which was then used as a means to close the centralized control loop determining the agitation mode in the fluidic environment. In the first study, we addressed the problem of the self-assembly of 3-cm-size floating passive modules. The design of the passive modules included single-state permanent magnetic latches that were installed in the 3D printed shells at proper distances so that the inter-module bonds could be reversible. With the dynamics of the system allowing for the process to be fully observable, we developed a dedicated computational framework guiding the SA process to achieve the target structure based on a model of the system. The framework provided real-time online model-based control through real-time visual tracking of the modules, updating the parameters of a Chemical Reaction Network (CRN) model and optimizing the control action, i.e., the environmental agitation mode. Our experimental results demonstrated that the best control generated by the framework consistently outperformed the random control scheme, as well as a fixed agitation mode scheme, achieving a target structure composed of four modules within a few minutes. In addition, this study validated the functionality of the interplay of the assembly and disassembly in distributed systems for efficient realization of SA. While the framework had the capability to reliably guide the process to self-assemble a specific target, the underlying modeling technique did not scale well with the size of the swarm and was also restricted in the case of partially observable systems. Both of the aforementioned conditions are common in the case of SA at the micro-scale where typically numerous building blocks are available and the highly noisy and erratic dynamics of the system makes the processes only partially observable. Our second study was characterized precisely by such conditions. Consequently, we adjusted our overall objectives and demonstrated the closed-loop control of the size of an aggregate rather than its precise shape. Using a similar approach to the one deployed at the centimeter scale, the SA of particles into clusters of a pre-set dimension was obtained through real-time high-speed tracking of the dynamics of the system, employing a dedicated image processing method, though still implemented in the very same tracking framework used before (SwisTrack), and guiding the system towards the target by applying proper environmental agitation favoring the assembly or disassembly of the microparticles. The microparticles were carefully designed and fabricated to allow for the specific connection and facilitating visual tracking. While in the case of the SA of passive modules, the SA process can only be influenced by the design of the physical characteristics of the modules and through centralized control, active modules allow for distributing the control in the system. Our third study was specifically dedicated to this idea and addressed the programmable SA of miniaturized floating robots where several copies of the target structure may be built in parallel through multiple concurrent local interactions. The active modules were endowed with on board processing power and custom-designed controllable electro-permanent magnetic latches, also used for local communication. Our experiments demonstrated how the SA process towards a specific target structure could be guided by the programmable behavior of the robots in a fully-distributed and, thus, scalable fashion using a behavior encoded in the embedded rule sets, with the environment also actively taking part in the process by providing feedback to the modules. Our experiments also characterized the system and showed that the modeling assumptions of the $M^{3}$ framework held for the system consisting of the Lily robots, thus allowing for further investigation and application of the framework. With the SA process being mainly guided by the behavior of the robots, the size of the underlying CRN model grew solely with the size of the rule set behavior rather than the size of the total possible configurations of sub-assemblies, making the problem tractable using the same method.

In the future, we plan to continue this line of research along several axes. In particular, we will investigate scenarios in which SA is realized through a combination of distributed and centralized control. We will study the problem of rule set design for the programmable SA of robots with the specific goal of deriving rules that allow the SA process to be as parallel as possible depending on the structure of the desired target. Additionally, we plan to develop a full multi-level modeling framework for the system: capture the dynamics of the system in a faithful robotics simulator and add microscopic and macroscopic modeling layers. Finally, we will deploy our experimental platform to conduct real robot experiments in large swarms of up to 100 Lily robots.
